# Preclinical Development of ARV-2001, an Intradermally Administered mRNA–Lipid Nanoparticle Immunotherapeutic for the Treatment of HPV-16-Positive Cervical High-Grade Squamous Intraepithelial Lesions

**DOI:** 10.3390/vaccines14080714

**Published:** 2026-08-19

**Authors:** Zhengxiang He, Huabin Zhu, Ju Hyeong Jeon, Jianzhu Chen, Gregory M. Glenn, Renhuan Xu

**Affiliations:** 1 Pinion Immunotherapeutics, North Bethesda, MD 20852, USA; 2 Department of Biology and Koch Institute for Integrative Cancer Research, Massachusetts Institute of Technology, Cambridge, MA 02139, USA

**Keywords:** HPV16, mRNA vaccine, cervical high-grade squamous intraepithelial lesion, intradermal injection, T-cell immunity, TC-1 tumor model

## Abstract

**Background/Objective:** Persistent infection with human papillomavirus type 16 (HPV-16) is the principal cause of cervical high-grade squamous intraepithelial lesions (cHSIL) and cervical cancer, yet the established treatments remain limited to ablative or excisional procedures that carry reproductive risk and do not eliminate the underlying infection. We report the preclinical development of ARV-2001, a messenger RNA (mRNA)–lipid nanoparticle (LNP) immunotherapeutic encoding mutated, non-oncogenic HPV-16 E6 and E7 fused to a SARS-CoV-2 spike S2 subdomain enriched in human CD4 helper epitopes, formulated in a novel cholesterol-derived ionizable lipid (ARV-T1). **Methods:** Interactions of ARV-2001-expressed antigens with p53 and retinoblastoma (Rb) were evaluated in human cervical carcinoma cell line C33A, in lentiviral constructs in primary human keratinocytes, and in soft-agar colony-formation assays. ARV-2001 was administrated by intramuscular (IM) or intradermal (ID) injection in naive mice or in the TC-1 tumor models. Tumor size and survival were monitored over time and tumor-infiltrated lymphocytes were characterized by flow cytometry. Intracellular cytokine staining and Elispot were used to evaluate immunogenicity. **Results:** In vitro, the mutated E6/E7–S2 antigen lost the ability to degrade p53, to deregulate the retinoblastoma (Rb) pathway, and to support anchorage-independent growth, suggesting abrogation of oncogenic activity. The S2 domain and imiquimod administration each augmented antitumor activity and intratumoral CD8^+^ T-cell infiltration while reducing myeloid-derived suppressor cells in the syngeneic HPV-16 E6/E7 TC-1 tumor models. In addition, ID administration of ARV-2001 into TC-1 tumor-bearing mice was superior to IM administration in terms of both tumor growth inhibition and survival. ID vaccination with ARV-2001 in mice consistently elicited a more potent E6/E7-specific T-cell response than the same dose given IM. Dose-escalation studies showed a dose-dependent T cell response against E6/E7 in ID-injected mice. **Conclusions:** This study supports future human evaluation of intradermally administrated ARV-2001 for treatment of HPV-16+ cHSIL in clinical trials.

## 1. Introduction

Cervical cancer remains a leading cause of cancer death among women worldwide and is almost exclusively attributable to persistent infection with high-risk human papillomavirus (HPV) genotypes, of which HPV-16 is the most prevalent and most oncogenic [1,2]. Although prophylactic HPV vaccines have markedly reduced incidence when administered before exposure, they have no effect on established infection, leaving screening and treatment of precancerous lesions as the only options for previously exposed individuals [3]. The immediate precursor of invasive squamous cell carcinoma is the cervical high-grade squamous intraepithelial lesion (cHSIL; cervical intraepithelial neoplasia grade 2 or 3, CIN2/3) [4].

The current standard of care for cHSIL is destructive or excisional removal of the cervical transformation zone, most commonly by loop electrosurgical excision procedure (LEEP) [5]. While effective at removing visible disease, these procedures do not address the underlying viral infection, are associated with persistent or recurrent disease in 5–27% of women, and carry reproductive consequences, including an approximately two-fold increase in preterm birth, in a population that is frequently of childbearing age [5,6]. HPV-16 in particular exhibits the lowest post-excisional clearance and the highest recurrence risk among high-risk genotypes [7]. A non-surgical, cervix-sparing therapy that clears HPV-16 and induces durable lesion regression therefore represents a substantial unmet need.

HPV-16 evades immune detection by persisting within keratinocytes with minimal viral protein expression and without cytolysis, thereby limiting the release of the pro-inflammatory signals that would normally prime an adaptive response [8]. While antibodies prevent primary infection, clearance of HPV-transformed cells requires effective T-cell-mediated immunity directed at the E6 and E7 oncoproteins [9]. Because E6 and E7 are obligate, constitutively expressed drivers of the transformed phenotype, E6 degrading p53 and E7 inactivating Rb, they are constrained from immune-escape mutation and constitute ideal therapeutic targets [10,11]. Clinical proof of concept that T-cell immunity against E6/E7 can drive regression of established HPV-16 lesions has been demonstrated with synthetic long-peptide vaccination in vulvar intraepithelial neoplasia, where clinical responses correlated with the magnitude of the vaccine-induced T-cell response [12,13], and with DNA-based immunotherapy in CIN2/3 [14]. Across therapeutic HPV vaccine trials, however, efficacy has been modest; a meta-analysis of phase II/III studies reported pooled CIN2/3 regression of 54% in vaccinated participants versus 27% in placebo recipients [15], underscoring both the validity of the approach and the opportunity to improve upon it.

Messenger RNA (mRNA)–lipid nanoparticle (LNP) platforms offer mechanistic advantages for this indication. Endogenous translation of the encoded antigen directly loads the major histocompatibility complex (MHC) class I pathway in professional antigen-presenting cells (APCs), generating potent CD8^+^ responses without the inefficient cross-presentation on which protein and DNA platforms rely, while the LNP supplies intrinsic innate-adjuvant activity [16]. We hypothesized that three additional design elements would further increase the probability of durable lesion clearance: (i) inclusion of a SARS-CoV-2 spike S2 subdomain rich in CD4 helper epitopes to recruit pre-existing memory T-cell help in a COVID-19–experienced population; (ii) topical imiquimod, a toll-like receptor (TLR) 7/8 agonist, applied to the cervix to remodel the immunosuppressive lesional microenvironment and recruit vaccine-primed T cells—a “prime-pull” strategy shown to establish tissue-resident memory at mucosal sites [17,18,19]; and (iii) intradermal (ID) administration to exploit the high density of dermal APCs while restricting systemic exposure [20,21].

Here, we describe the integrated preclinical development of ARV-2001, an mRNA–LNP immunotherapeutic encoding mutated HPV-16 E6/E7 fused to S2 and formulated in the proprietary ionizable lipid ARV-T1, for the treatment of HPV-16-positive cervical high-grade squamous intraepithelial lesions. These data formed the basis of an Investigational New Drug (IND) application cleared by the U.S. Food and Drug Administration (FDA) in January 2026 and the design of the first-in-human study PIN-001 in future.

## 2. Materials and Methods

### 2.1. Animal Studies

Six- to eight-week-old female BALB/c mice (Strain: 000651) and C57BL/6 mice (Strain: 000664) were purchased from Jackson Lab and maintained at Pinion Immunotherapeutics animal facility. Animals were randomly assigned to different groups. All animal experiments in this study were approved by the Institutional Animal Care and Use Committee of Pinion Immunotherapeutics and were performed in accordance with the approved guidelines for animal experimentation.

### 2.2. Reagents for LNP Formulation

Cholesterol (plant-derived, Cat: 700100P) 1,2-distearoyl-sn-glycero-3-phosphocholine (DSPC, Cat: 850365) and 1,2-dimyristoyl-snglycero-3-phosphoethanolamine-N-[methoxy(polyethylene glycol)-2000] (DMG-PEG2000, Cat: 880151P) were obtained from Avanti Polar Lipids (Alabaster, AL, USA). The proprietary ionizable lipid ARV-T1 was synthesized according to previously reported procedures [22].

### 2.3. In Vitro mRNA Transcription (IVT) and LNP Formulation

The linear dsDNA of EGFP-firefly luciferase (EGFP-Luc) and HPV16 mutant E6 (F9V, C70G and I135 T) and E7 (H2P, C24G and E26G) fused with CD4 epitopes from the SARS-CoV2 Delta variant spike protein (S2) (E6-E7-S2) were synthesized from GenScript. Corresponding plasmids for templates of IVT were generated from pUC57 plasmid, which contains a CleanCap AG T7 promoter [23], 5′-UTR, 3′-UTR, and polyA tail. All mRNA syntheses, LNP formation, and characterization were conducted as previously reported [22,24]. Briefly, the linearized plasmids were in vitro transcribed by using T7 RNA polymerase (low dsRNA) (Yeasen, Gaithersburg, MD, USA, Cat:10628ES10) with CleanCap Reagent AG (TriLink, San Diego, CA, USA, Cat: N-7113) for co-transcriptional capping. The mRNA was purified by Pierce™ Protein Concentrators PES, 100 K MWCO (Thermo Fisher Scientific, Waltham, MA, USA, Cat: 88533). The integrity and purity of mRNA were assessed by NanoDrop spectrophotometer (Thermo Fisher Scientific) and electrophoresis. Purified mRNAs were encapsulated in LNP formulation. A INano L system (Micro&Nano, Shanghai, China) was used for formulation, maintaining a specific ratio (1:3, *v*/*v*) between the organic (ethanol) and aqueous phases (0.13 µg/µL mRNA in 50 mM citrate buffer, pH 4.0). The lipids were mixed in ethanol with the molar percentage ratio as follows: 50% ARV-T1 (ionizable lipid), 10% DSPC, 38.5% cholesterol, and 1.5% DMG-PEG2000. The formulations were tested for particle size and distribution using a dynamic light scattering (DLS) instrument (Malvern, Worcestershire, UK). Encapsulation efficiency of mRNA-loaded LNPs were evaluated using Ribogreen assay (Thermo Fisher Scientific, Cat: R11490).

### 2.4. In Vitro Assay of mRNA and mRNA-Loaded LNPs

The HEK293T cell line (CRL-3216) was obtained from the American Type Culture Collection (ATCC). Cells were seeded in 12-well plates at a concentration of 2.5 × 10^5^ cells per well in Dulbecco’s modified Eagle’s medium (DMEM) (Gibco, New York, NY, USA, Cat: 11965092) with 10% fetal bovine serum (Gibco, Cat: A5256701) one day prior to the transfection and incubated at 37 °C in a humidified atmosphere containing 5% CO_2_. For mRNA transfection, Lipofectamine MessengerMAX Transfection Reagent (Invitrogen, Waltham, MA, USA, Cat: LMRNA001) and LNPs loaded with corresponding mRNA were added dropwise to the cells. Non-treated wells were run in parallel as non-transfected control. Following 48 h of incubation, cells were collected for examining corresponding mRNA encoded protein expression by Western blot or fluorescence microscopy.

### 2.5. Western Blot

Western blotting was used for detecting E6-E7-S2 fusion protein expression by ARV-2001. Briefly, 20 µg of protein from the whole cell lysate was separated by SDS-PAGE (Thermo Fisher Scientific, Cat: NP0322BOX) and transferred to a PVDF membrane (Thermo Fisher Scientific, Cat: IB24002). The PVDF membrane was then incubated with the primary HPV16 E7 Antibody (Santa Cruz, Dallas, TX, USA, Cat: sc-51951), followed by incubation with Anti-mouse IgG(H + L)-HRP (SouthernBiotech, Birmingham, AL, USA, Cat: 1036-05). Anti-beta-actin HRP antibody (Santa Cruz, Cat: sc-47778 HRP) was used as protein loading control. The membrane was developed using SuperSignal™ West Pico PLUS Chemiluminescent Substrate (Thermo Fisher Scientific, Cat: 34580), and the images were captured using a Bio-imager (PerkinElmer, Shelton, CT, USA).

### 2.6. Oncogenicity (p53/Rb and Anchorage-Independent Growth) Assays

Interactions of ARV-2001-expressed antigens with p53 and retinoblastoma (Rb) were evaluated in human cervical carcinoma cell line C33A (HTB-31, ATCC), in lentiviral constructs in primary human keratinocytes (ATCC, Cat: PCS-200 010), and in soft-agar colony-formation assays. Wild-type E6/E7 served as positive controls for p53 degradation, Rb-pathway deregulation, and anchorage-independent growth; mutated E6/E7 antigens, including the E6/E7–S2 fusion, were compared in parallel.

The LNP-mRNAs encoding wild-type E6 (WT E6), WT E6-E7 fused with S2, and ARV-2001 were transfected into C33A cell line. Cells were collected 72 h after transfection, and the whole cell extracts (50 μg protein/well) were used to perform Western blotting for detection of the target antigen. Human papillomavirus type 16 E6 antibody (GeneTex, Irvine, CA, USA, Cat: GTX132686) was used for E6 (17 kDa) or E6/E7 fused with S2 (50 kDa) expression and purified anti-p53 Antibody (BioLegend, San Diego, CA, USA, Cat: 645702) was used for endogenous p53 expression in transduced C33A cells.

Pre-made lentivirus expressing WT E6-E7, mutant E6, mutant E7 and mutant E6-E7 fused with S2 were customer made in GenTarget, Inc. (San Diego, CA, USA). The lentivirus transduction into primary human keratinocytes (ATCC, Cat: PCS-200 010) was performed according to the manufacturer’s instructions. Briefly, cells were seeded at 0.5 × 10^5^/mL × 0.5 mL per well in 24-well plates. The pre-made lentiviral stocks were thawed at room temperature and 100 μL of virus was added into each well. 72 h after transduction, RFP was checked by flow cytometry to calculate the transduction rate. For the Rb expression, transduced cells were performed by intracellular staining. In brief, cells were stained with Zombie Aqua Fixable viability dye (BioLegend, Cat: 423102) in PBS for 20 min at room temperature (RT). Cells were then fixed and permeabilized with Foxp3/Transcription Factor Staining Buffer Set (Invitrogen, Cat: 50-112-8857). Intracellular staining was performed with anti-under-phospho Rb antibodies (Miltenyi Biotech, Auburn, CA, USA, Cat: 130-110-606) at 4 °C for 2 h. In parallel, REA control antibody, human IgG1 (Miltenyi Biotech, Cat: 130-113-446) was used as an isotype control. Cells were washed twice with permeabilization buffer and resuspended in flow cytometry buffer. The cells were analyzed by flow cytometry.

For the clonogenic capacity assay, a Cell Transformation Assay Kit (Colorimetric) (Abcam, Cambridge, UK, Cat: ab235698) was used following the manufacturer’s recommendations to detect lentivirus-transduced transforming properties in primary keratinocytes. Briefly, equal numbers (1 × 10^4^ cells) of transduced primary keratinocytes were seeded for the cell transformation assay (d0). After culture in 3D soft agar for 7 days (d7), the cell numbers were quantified by spectrophotometry at 450 nm after addition of the WST Working Solution provided in the kit.

### 2.7. Injection of LNP-mRNA into Mice

For ID immunizations, vaccine was injected into the ear (immunogenicity and efficacy assay) or dorsal skin (biodistribution assay) in a volume of 20 µL per injection with a 28-gauge needle (Cardinal Health, Dublin, OH, USA, Cat: 8881511110). Briefly, the needle was inserted parallel to the plane of the skin. 20 µL of LNPs-mRNA at indicated concentration were injected slowly and the needle slowly withdrawn 5 s after the injection was completed. The formulation of a small and round skin welt indicates properly performed for ID injection. For the IM administration group, 20 µL of LNP-mRNA was injected into the quadriceps of mice with a 30-gauge insulin syringe.

### 2.8. Bioluminescence (In Vivo Imaging)

At indicated time points after the injection of the LNPs-mRNA, Balb/c mice were intraperitoneally (IP) injected with 0.2 mL D-luciferin (10 mg/mL in DPBS) (GoldBio, Olivette, MO, USA, Cat: LUCNA-1G). Mice were anesthetized in a ventilated anesthesia chamber with 1.5% isoflurane in oxygen and imaged 10 min after the injection using an IVIS Spectrum Imaging System (PerkinElmer). Post-imaging, the acquired signal was quantified utilizing Living Image^®^ 4.5.5 Software (IVIS Imaging Systems) and expressed as total flux (radiance per sec).

### 2.9. Tumor Inoculation and Tumor Size Measurement

The female C57BL/6 mice were inoculated with TC-1 tumor (2 × 10^5^ cells/mouse, subcutaneously injection) on the right flank. Tumor sizes were measured unblinded using an electronic caliper twice per week for calculating tumor volumes using the equation (width × width × length)/2. On day 10 post-tumor inoculation, mice with tumor size about 100 mm^3^ were separated into different treatment groups (tumor volumes were balanced before treatment). Animals were euthanized when mice either showed signs of suffering, or the average tumor volume exceeded 2000 mm^3^.

### 2.10. Intracellular Cytokine Staining

Single-cell suspensions were made from spleens by homogenizing through 70 µm cell strainers using the plunger of a syringe, as described previously [25]. Erythrocytes were lysed with erythrocyte lysis buffer (ACK lysis buffer) (Lonza, Basel, Switzerland, Cat: BP10-548E) for 5 min and resuspended in RPMI 1640 medium (Gibco, Cat: 11875093) with 10% fetal bovine serum (Gibco, Cat: A5670801) and 1% 2-mercaptoethanol (Gibco, Cat: 21985023). Then, 2 × 10^5^ splenocytes per well were seeded into round-bottom 96-well plate (Corning, Corning, NY, USA, Cat: 3788) and stimulated with 1 µg/mL HPV16 E6 peptide pool and 1 µg/mL E7 peptide mix (JPT, Berlin, Germany, Cat: PM-HPV16-E6 and PM-HPV16-E7) or cell activation cocktail (Biolegend, San Diego, CA, USA, Cat: 423302) or medium alone in the presence of protein transporter inhibitor cocktail (Thermo Fisher Scientific, Cat: 00-4980-03) at 37 °C overnight. Cells were then stained with Zombie Aqua Fixable viability dye (BioLegend, Cat: 423101) in PBS for 20 min at room temperature (RT). Cells were subsequently incubated with purified anti-mouse CD16/32 Antibody (BioLegend, Cat: 101302) in flow cytometry buffer (PBS supplemented with 2% heated inactivated FBS) for blocking non-specific binding of immunoglobulin at 4 °C for 30 min. Then, cells were stained with surface markers (anti-CD45, CD3, CD4, CD8) at 4 °C for 30 min. Cells were fixed and permeabilized using an Intracellular Fixation and Permeabilization Buffer Set (Thermo Fisher Scientific, Cat: 88-8824-00) according to the manufacturer’s instructions. Intracellular staining was performed with anti-INFγ and TNFα in 1X permeabilization buffer at 4 °C for 1 h. Cells were washed twice with permeabilization buffer and resuspended in flow cytometry buffer. The cells were analyzed by flow cytometry using MACSQuant 16 (Miltenyi Biotec). Data were analyzed with FlowJo software v10 (TreeStar, Ashland, OR, USA). Antibodies used were as follows: AF780 CD45 (Thermo Fisher Scientific, clone 30-F11, Cat: 47-0451-82), PE-Cy7 CD3 (BioLegend, clone 17A2, Cat: 100220), FITC CD4 (BioLegend, clone GK1.5, Cat: 100406), BV605 CD8 (BioLegend, clone 53-6.7, Cat: 100743), PE/Dazzle 594 INFγ (BioLegend, clone XMG1.2, Cat: 505846) and APC TNFα (Thermo Fisher Scientific, clone MP6-XT22, Cat: 17-7321-82).

### 2.11. Enzyme-Linked Immune Absorbent Spot (ELISPOT) Assay

IFNγ ELISPOT assays were performed as described in our previous publication [24]. Briefly, a 96-well ELISPOT plate (Millipore, Burlington, MA, USA, Cat: MSIPS4510) was coated with 10 µg/mL IFNγ antibody (Biolegend, clone AN18, Cat: 517902) at 4 °C overnight. Splenocytes were plated at 2 × 10^5^ cells/well and co-cultured with 1 µg/mL HPV16 E6 peptide pool and 1 µg/mL E7 peptide pool (JPT, Cat: PM-HPV16-E6 and PM-HPV16-E7) in medium or medium alone in a total volume of 200 µL/well T-cell media for 48 h at 37 °C in 5% CO_2_. The plates were incubated with detection antibodies Biotin-IFNγ (Biolegend, clone R46A2, Cat: 505714) and Streptavidin-HRP (Biolegend, Cat: 405210) at RT for 1–2 h, respectively. The plates were developed with 50 µL/well AEC development solution (BD biosciences, Franklin Lakes, NJ, USA, Cat: 551015) for 4–10 min. Color development was stopped by washing under running tap water. After air drying, colored spots were counted using automated ELISPOT reader system (CTL Analyzers) with ImmunoSpot software Version 7.0.21.0 (Shaker Heights, OH, USA). The average of spot-forming cells (SFC) was adjusted to 1 × 10^6^ splenocytes for data display.

### 2.12. Statistical Analysis

The data are shown as the mean values ± SEMs throughout. No statistical method was used to predetermine the sample size. Statistical analyses were performed with GraphPad Prism 10 software (GraphPad Prism 10, Boston, MA, USA). For the comparison of multiple data sets, nonparametric Mann–Whitney test was used, whereas the two-tailed unpaired Student’s *t*-test was used for comparing two groups. In the mouse tumor model, tumor sizes were compared using ANOVA with the Bonferroni post hoc test, and survival rates were analyzed with the log-rank test. Differences were considered significant when *p* < 0.05, and levels of significance are specified throughout the figure legends.

## 3. Results

### 3.1. ARV-2001 Antigen Design and Abrogation of E6/E7 Oncogenic Activity

**Figure 1:**
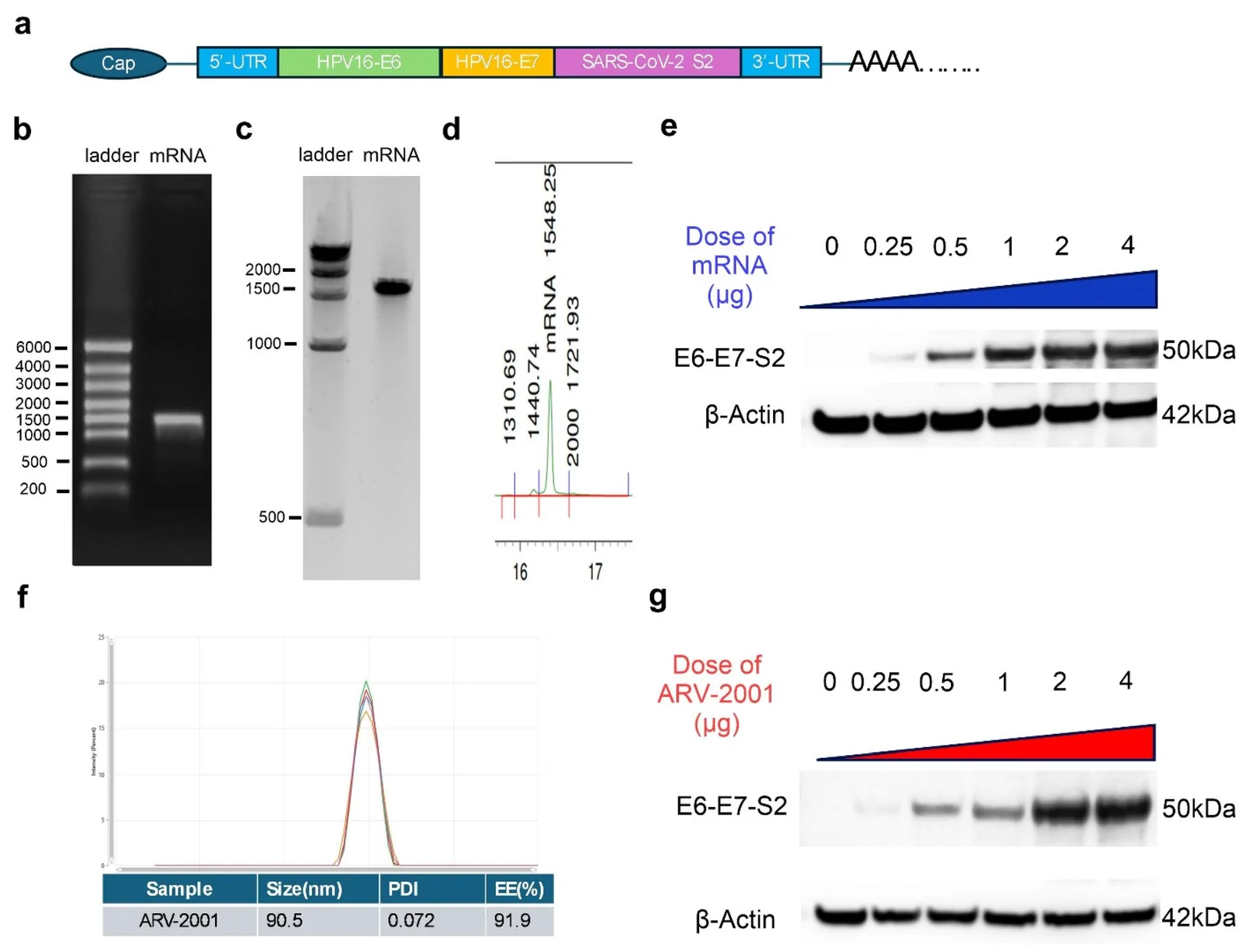
Characterization and in vitro potency of ARV-2001, an LNP formulated mRNA encoding E6-E7-S2. (**a**) Schematic of ARV-2001 mRNA encoding mutated HPV-16 E6 (F9V, C70G and I135 T) and E7 (H2P, C24G and E26G) fused to the CD4-epitope-enriched SARS-CoV-2 S2 subdomain. (**b**–**d**) Quality of the in vitro-transcribed mRNA encoding E6-E7-S2 assessed by agarose gel electrophoresis (**b**), urea-TBE PAGE electrophoresis followed by Gel Blue staining (**c**) and capillary gel electrophoresis with laser-induced fluorescence (CGE-LIF) (**d**) confirming the integrity and size of mRNA. (**e**) In vitro expression of the fusion protein from mRNA transfected by Lipofectamine MessengerMAX Transfection Reagent. Western blot analysis of fusion protein expression in mRNA-transfected cells probed with an anti-HPV16 E7 antibody. β-actin was used as a loading control. (**f**) Physicochemical properties and encapsulation efficiency (EE) of ARV-2001. The size and PDI were characterized by dynamic light scattering and EE(encapsulation efficiency) by RiboGreen method. (**g**) Western blot detection of the E6-E7-S2 fusion protein in ARV-2001-transfected cells 48 h post-transfection, with β-actin as a loading control. Results in (**e**–**g**) are representative of three independent experiments.

ARV-2001 encodes mutated HPV-16 E6 (F9V, C70G and I135 T) [26] and E7 (H2P, C24G and E26G) [26,27] fused to the CD4-epitope-enriched SARS-CoV-2 spike S2 subdomain (Figure 1a). In vitro transcription yielded full-length mRNA of the expected size (Figure 1b–d). To verify the in vitro expression, we conducted Western blotting on HEK293T cells post-mRNA transfection, using Lipofectamine MessengerMAX Transfection Reagent. We observed specific bands with molecular weights of 50 kDa for fusion protein, consistent with its expected size, in a dose-dependent manner (Figure 1e and Figure S1). We have previously demonstrated that the LNPs formulated with self-developed ARV-T1 showed significantly increased mRNA delivery both in vitro and in vivo, markedly increased protein expression, and demonstrated high potency in inducing antibody responses against SARS-CoV2 in mice [22]. Here, we used ARV-T1 as ionizable lipid for HPV16 therapeutic mRNA delivery. The LNP encapsulating mRNA encoding E6-E7-S2 fusion protein (ARV-2001) had a size of 90.5 nm with a PDI of 0.072, and the formulation encapsulation efficacy was 91.9% (Figure 1f). HEK293T cells were transfected with ARV-2001 for 48 h, the cells were lysed, and Western blot analysis was performed using a HPV16 E7 antibody. A 50 kDa band was detected that was consistent with the expected size of E6-E7-S2 fusion protein (Figure 1g). In addition, dose-dependent antigen expression levels by ARV-2001 were observed in vitro (Figure 1g and Figure S2).

**Figure 2:**
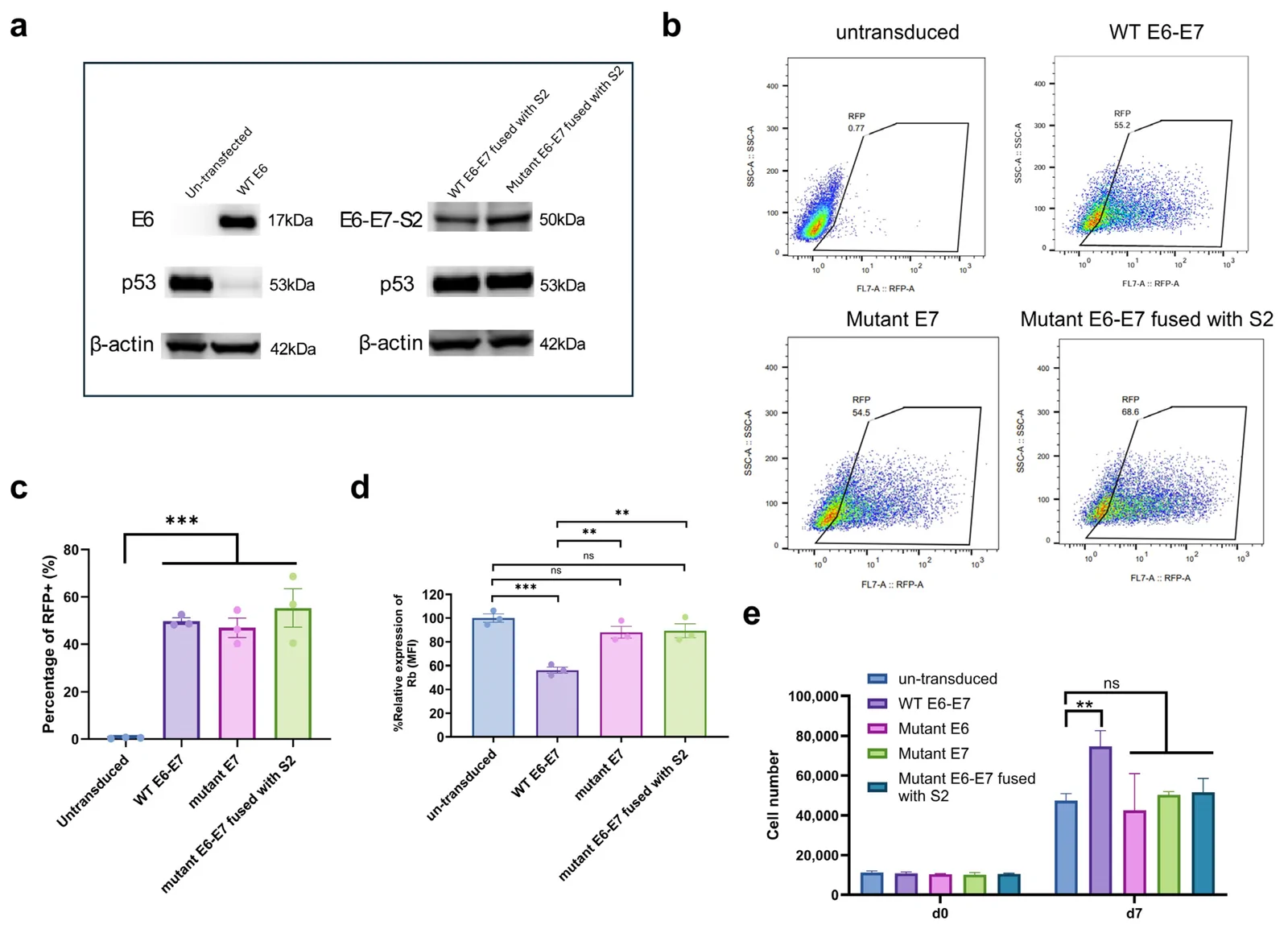
Oncogenicity assays (p53/Rb and anchorage-independent growth). (**a**) Interaction of the proteins translated from WT E6, mutant E6-E7-S2, WT E6-E7-S2 with p53 in the C33A cell line. Ten picomoles of the indicated mRNA-LNPs were transfected into C33A cells. Cells were collected 72 h after transfection, and the whole cell extracts (50 μg protein/well) were analyzed by Western blot for detection of the target antigen. β-actin was used as a loading control. Results are representative of three independent experiments. (**b**–**d**) Interaction of the proteins translated from WT E6-E7, mutant E7 and mutant E6-E7 fused to S2 with Rb in primary keratinocytes. (**b**) Representative FACS plots showing percentage of RFP+ cells in lentivirus-transduced vs. untransduced primary human keratinocytes. (**c**) Quantification of the percentage of RFP^+^ cells in lentivirus-transduced vs. untransduced primary human keratinocytes. (**d**) Mean fluorescence intensity (MFI) of underphosphorylated Rb expression in lentivirus-transduced keratinocytes. Underphosphorylated Rb expression was determined by intracellular staining of both un-transduced and transduced RFP^+^ keratinocytes. (**e**) Transforming activity measured with Cell Transformation Assay Kit (Colorimetric) (Abcam, ab235698). Equal numbers of transduced primary keratinocytes (1 × 10^4^ cells) were seeded into plates. After 7 days, cell numbers were quantified by spectrophotometry absorbance at 450 nm. Statistical analysis using two-tailed unpaired Student’s *t*-test for (**c**,**e**). Statistical analysis using nonparametric Mann–Whitney test for (**d**). ** *p* < 0.01, *** *p* < 0.001; ns, not significant.

To verify that mutation eliminated oncogenic function, the E6/E7 antigens were tested against their canonical targets. The translated proteins from mRNA-LNP formulations encoding WT E6, WT E6-E7 fused with S2, and mutant E6-E7 fused with S2 (from mRNA-2001) were analyzed for their ability to dysregulate cells in vitro. After transfection with the mRNA-LNP formulations, the cells were examined for expression of p53 tumor suppresser proteins, which are known to be reduced by the WT HPV-16 E6 protein [28]. As expected, p53 protein expression was reduced in C33A cells expressing WT E6 (positive control), but not in cells expressing mutant E6-E7 fused with S2. Notably, the protein expressed by WT E6 E7 fused with S2 did not reduce p53 expression either (Figure 2a and Figure S3). It is possible that the E6/E7 fusion approach itself diminished the ability of WT E6 to initiate degradation of p53 proteins. Also, since the E6-E7-WT-S2 fusion protein and the E6(mutant)-E7(mutant)-S2 fusion had the E6 gene fused to the E7 gene, it is possible that the mutations present in the mutated E6 gene and the E6 fusion construction both contributed to the ARV-2001 vaccine not reducing the p53 content in the cells.

HPV-16 E7 is known to promote the degradation of Rb [29]. We cloned the DNA sequences encoding HPV16 WT E6-E7 (positive control), mutant E7 and mutant E6-E7 fused with S2 (identical sequences to that of ARV-2001) into lentivirus (red fluorescent protein as a reporter). Primary human keratinocytes were infected with the lentiviral constructs and the expression of the target proteins was measured via RFP cell counts in transduced keratinocytes by flow cytometry 3 days later. No significant difference was seen in RFP expression in the individual target-expressing lentivirus-transduced keratinocytes (Figure 2b,c), indicating similar expression levels in transduced keratinocytes. As expected, lentivirus in keratinocytes expressing WT E6-E7 (positive control) showed decreased active form of Rb expression by flow cytometry compared to those in un-transduced keratinocytes (Figure 2d). No significant differences were detected in the expression of active form of Rb (Figure 2d) in lentivirus expressing mutant E6-E7 fused with S2 keratinocytes (RFP+ population in Figure 2b,c).

The capacity of HPV-16 E6 and E7 to transform cells is related to the ability to disable the function of the tumor suppresser proteins p53 and Rb. In the soft-agar assay, transformed cells gain the ability to proliferate throughout the soft agarose gel without attaching to a surface and tend to form spheroid-like clumps of cells (colonies). The Cell Transformation Assay Kit (ab235698) produced a colorimetric (450 nm) readout, providing a surrogate transformation assay. At 7 days, only lentivirus expressing WT E6-E7 (positive control) transduced keratinocytes, while mutant E6-E7 fused with S2 (i.e., ARV-2001) did not (Figure 2e).

In conclusion, wild-type E6 promoted p53 degradation, and wild-type E7 deregulated the Rb pathway and supported anchorage-independent growth in soft agar. In contrast, the mutated E6 and E7 antigens—including the E6/E7–S2 fusion—failed to degrade p53, did not deregulate the Rb pathway, and did not form colonies in soft agar. These results confirm that the clinical antigen retains immunogenic E6/E7 sequence but lack the transforming activity of the native oncoproteins, supporting its use as a non-oncogenic tumor-specific antigen.

### 3.2. Prior Spike Immunization Enhances Immune Response to the S2-Containing Construct ARV-2001

**Figure 3:**
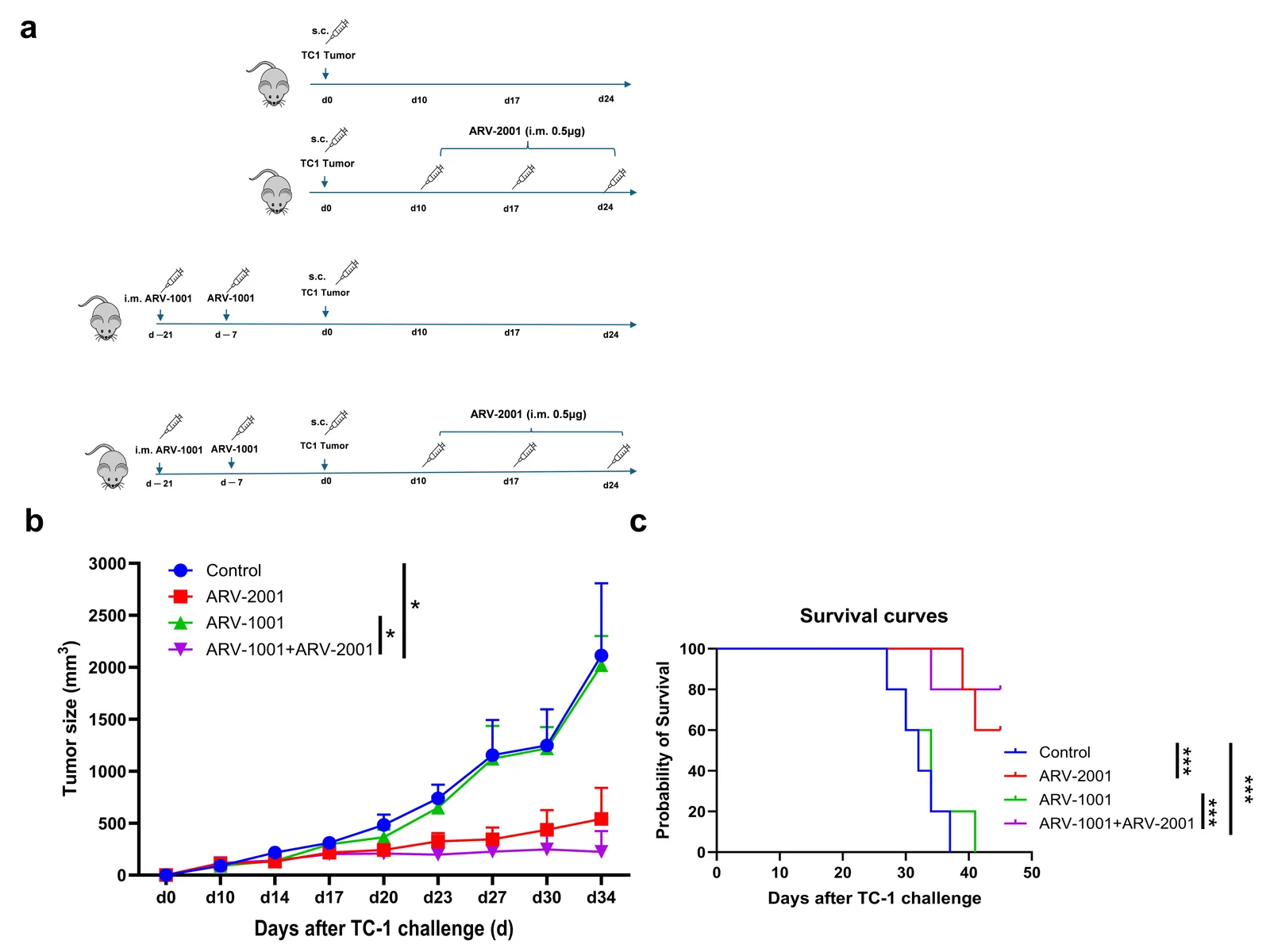
Prior spike immunization enhances antitumor activity of the S2-containing construct ARV-2001 in the TC-1 tumor models. (**a**) Study design. Two groups of mice were pre-vaccinated for two doses with ARV-1001 (day −21 and −7), and other two groups of mice were untreated. TC-1 tumor cells were inoculated subcutaneously (s.c.) on day 0 for all groups; by day 10 the average tumor size reached 100 mm^3^. Mice were then immunized weekly for three doses (day 10, 17 and 24) with ARV-2001. Tumor growth (**b**) and survival (**c**) were monitored over time. *n* = 5 mice/group. Statistical analysis using ANOVA with Bonferroni post hoc test for (**b**). Statistical analysis using a log-rank test for (**c**). * *p* < 0.05; *** *p* < 0.001.

TC-1 is a mouse cancer cell line derived from lung epithelial cells transformed with the oncogenic HPV-16 E6 and E7 genes [30]. Thus, the TC-1 tumor model is a well-established in vivo preclinical tool for testing therapeutic vaccines against HPV-16, given that no mouse mode of HSIL itself exists. To test whether the S2 subdomain can rapidly activate pre-existing memory T cells to promote CD8 T cell function and antitumor activity, mice were pre-vaccinated with ARV-1001 (an mRNA encoding the SARS-CoV-2 full-length spike) (day −21 and −7) before receiving three subtherapeutic doses of ARV-2001 (day 10, 17 and 24) (Figure 3a). Pre-vaccination of ARV-1001 followed by 0.5 µg ARV-2001 significantly inhibited TC-1 tumor growth relative to the control (Figure 3b,c). In a separate study to determine whether S2 contribute to enhance tumor-infiltrating leukocytes, pre-vaccination with ARV-1001 prior to two subtherapeutic doses of ARV-2001 produced significantly greater tumor reduction than ARV-2001 alone (Figure 3b), accompanied by a significant increase intratumoral CD45^+^ leukocyte infiltration relative to untreated controls (Figures S4 and S5). These findings indicate that engaging pre-existing memory T cells through the S2 domain amplified E6/E7-directed antitumor activity and lesion infiltration, supporting inclusion of the S2 fusion in the clinical construct.

### 3.3. Imiquimod Enhances Efficacy and Intratumoral CD8^+^ Infiltration (Prime-Pull)

The prime-pull hypothesis, referring to systemic priming of E6/E7-specific T cells combined with local innate activation to recruit them to the lesion, was tested using imiquimod (IMQ) (InvivoGen, San Diego, CA, USA, Cat: R837) (Figure S6a). Intratumoral IMQ alone and a subtherapeutic dose of ARV-2001 alone each prolonged survival relative to the control (Figure S6b,c), whereas the combination significantly inhibited tumor growth and prolonged survival relative to both the control and IMQ alone (Figure S6b,c). Mechanistically, the combination increased the proportion of intratumoral CD45^+^ leukocytes and CD4^+^ T cells relative to the control and increased CD8^+^ T cells relative to IMQ alone (Figures S4 and S7). These data demonstrate greater anti-tumor activity of the combination under the tested conditions.

### 3.4. Biodistribution of mRNA-LNP Administrated by the IM vs. ID Route

To evaluate the distribution of mRNA-encoded protein expression, we used an intravital bioluminescence assay with an in vivo imaging system (IVIS), which reported a surrogate protein rather than the vaccine antigen [31]. We formulated EGFP-luciferase mRNA-LNP using ARV-T1 ionizable lipid (Figure S8) and administered it by IM or ID into BALB/c mice at 0.1 μg, 1 μg and 5 μg (Figure S9a–c). The luciferase expressions at the injection site of IM injection at low dose (0.1 μg) and the medium dose (1 μg) were higher than that of ID injection (Figure S9a–c). At 6 h, luciferase expressions at the injection site was detected by both routes but was higher for IM than ID at low dose (0.1 μg) and the medium dose (1 μg), with no significant difference at 5 μg (Figure S9a–c). These findings are consistent with reports [32,33] that at early time point of post-injection (6 h), IM delivery of LNP generally results in higher protein expression than ID delivery, owing to the different cell types targeted. Expression was dose-dependent for both routes, confirming success in vivo expression and properly performed injection.

Next, we compared mRNA-encoded luciferase kinetic expression (6, 24 and 120 h post-injection) at the injection site by IM or ID administration at various doses of mRNA. In contrast to luciferase expression at 6 h post-injection, at 24 h post-injection, luciferase expression levels at injection site of ID injection were lower than those of IM injection only at high dose (5 μg) administration (Figure S9c), but not at low (0.1 μg) or medium (1 μg) doses of mRNA administrated (Figure S9a,b). In addition, both IM and ID groups showed very little to no detectable expression level of luciferase at 120 h post-injection of all test doses (Figure S9a–c). More importantly, the luciferase expression at injection site maintained similar expression levels 24 h post-IM injection regardless of the dose administrated, whereas lower luciferase expression levels were observed at 24 h post-ID injection at a high dose (5 μg) of mRNA (Figure S9c). Kinetically, injection site expression at 24 h was lower for ID than IM only at 5 μg (Figure S9c). and by 120 h was near-undetectable for both routes at all doses (Figure S9a–c).

Injected LNPs may enter into the bloodstream and reach the liver, resulting in off-target expression in the liver that may drive side effects and modulate immunity [34]. Therefore, the kinetic expression of mRNA-encoded luciferase in the liver of animals injected by IM or ID was also analyzed (Figure S9d–f). As expected, ID injection showed less luciferase expressions in the liver at very early time point (6 h) than the IM injection group regardless of which doses of mRNA were administrated (Figure S9d–f). By 24 h post-injection, hepatic luciferase was not detectable in the ID group at 5 μg, whereas a clear signal persisted in the IM group (Figure S9f). Thus, unlike IM, high-dose (5 μg) ID delivery minimized off-target expression and produced little to no detectable hepatic expression within one day.

### 3.5. In Vivo Anti-Tumor Efficacy of ARV-2001 Administrated by the IM vs. ID Route

**Figure 4:**
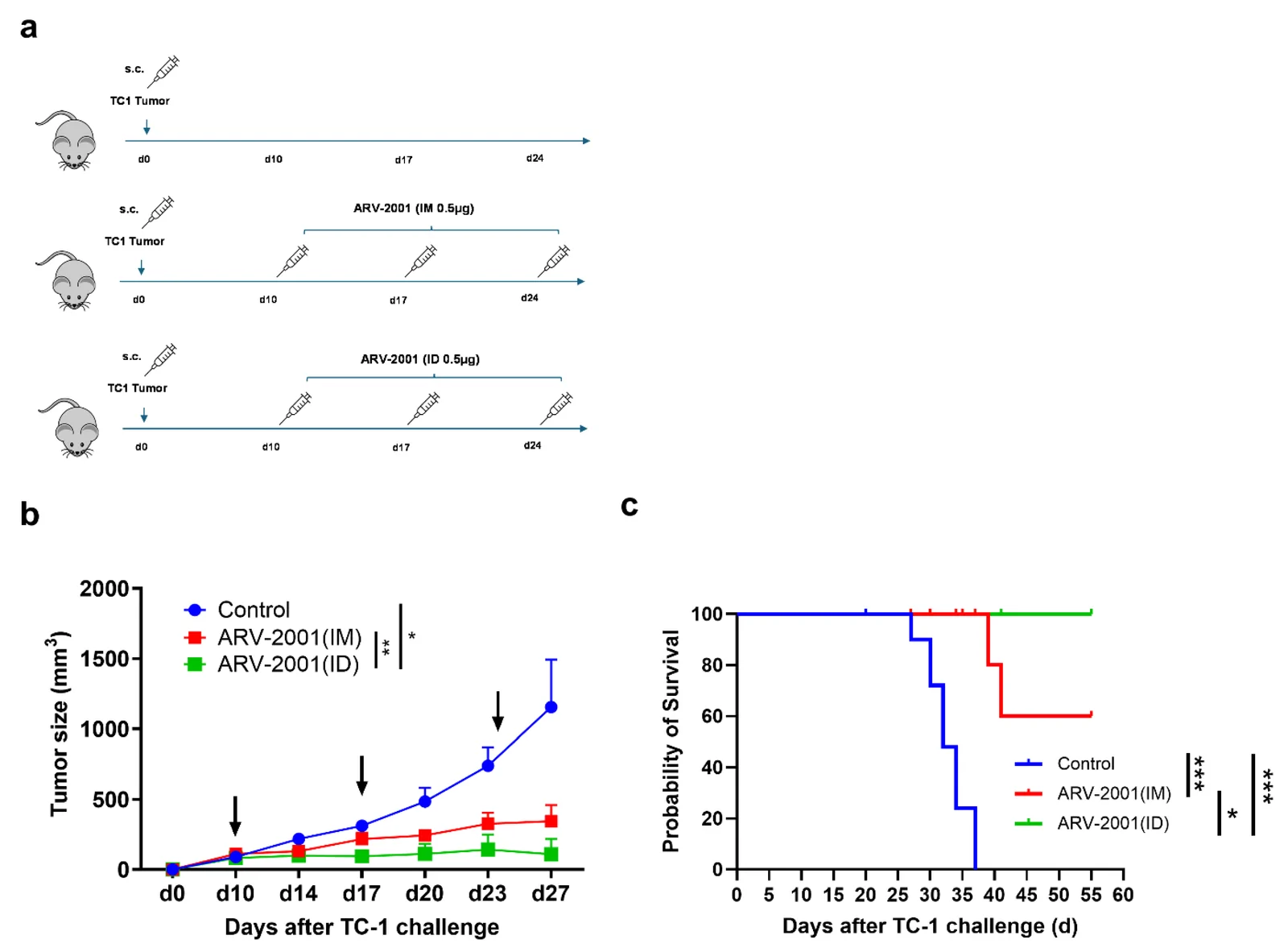
Therapeutic effect of ARV-2001 given ID versus IM in the TC-1 tumor model. (**a**) Schematic of the experimental procedure for ARV-2001 anti-tumor efficacy in mice. The average volume reached approximately 100 mm^3^ by 10 days after tumor inoculation. Mice in the control group were untreated. The treatment groups were vaccinated with ARV-2001 by either IM or ID injection weekly for three doses beginning on day 10. Tumor growth (**b**) and survival (**c**) were monitored and shown over time. The arrows indicate injection of vaccines. *n* = 5 mice/group. Statistical analysis using ANOVA with Bonferroni post hoc test for (**b**). Statistical analysis using a log-rank test for (**c**). * *p* < 0.05; ** *p* < 0.01; *** *p* < 0.001.

To compare the anti-tumor efficacy of ARV-2001 administrated by IM and ID injections, all groups were subcutaneously injected with TC-1 tumor cells on day 0. Ten days after tumor inoculation, mice in the ARV-2001 treated group were vaccinated with LNP loading mRNA encoding E6-E7-S2 weekly for three doses by IM or ID injection at d10, d17 and d24 (Figure 4a). The results for both administration routes showed robust tumor growth inhibition/regression and prolonged survival in mice (Figure 4b,c). Importantly, ID injection was superior to IM injection at the same dose of vaccine with respect to anti-tumor effects (Figure 4b,c). This is consistent with recent findings by Prins et al. [20], who observed that ID delivery of a COVID-19 mRNA vaccine was approximately five times more potent in inducing neutralizing antibodies than the IM route. We hypothesized that fast protein processing with ID delivery (Figure S9), which likely reflects the abundance of APCs in the dermis [35], with rapid ability to process the RNA-translated protein following ID injection.

### 3.6. T Cell Responses to ARV-2001 Administrated by the IM vs. ID Route

**Figure 5:**
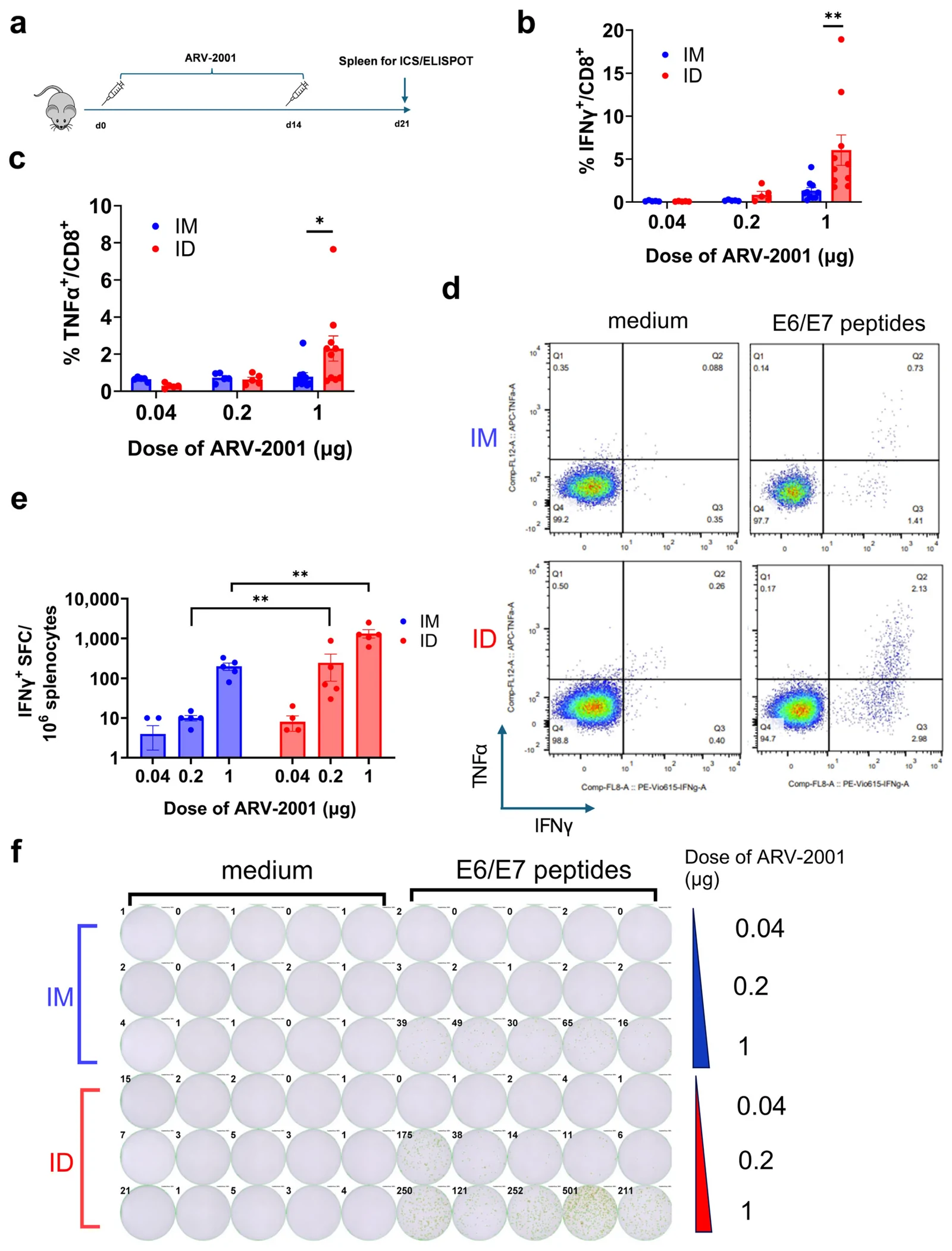
Immunogenicity of ARV-2001 given ID versus IM in naive mice. (**a**) Schematic of the experimental procedure. (**b**,**c**) Percentage of IFNγ^+^ (**b**) and TNFα^+^ CD8^+^ T cells (**c**) in the spleen after stimulations with E6/E7 peptides pool measured by ICS. (**d**) Representative FACS plots (gated on live CD45^+^CD3^+^CD8^+^) showing IFNγ and TNFα expression in splenic CD8^+^ T cells in mice immunized with 1-µg ARV-2001. (**e**,**f**) ELISPOT analysis of IFNγ producing cells s elicited by ARV-2001 administrated by ID versus IM injection. In (**b**,**c**,**e**), each dot indicates an individual mouse. Statistical analysis using two-tailed unpaired Student’s *t*-test for (**b**,**c**,**e**). * *p* < 0.05; ** *p* < 0.01.

We compared the cellular immune response induced by ARV-2001 in naive mice given IM or ID injection. The immunization scheme was as shown in Figure 5a. Splenocytes were collected 7 days after the last vaccination, and intracellular cytokines staining (ICS) was performed to determine the secreted effector cytokines IFNγ and TNFα responses to stimulation with a peptide pool spanning HPV16 E6/E7 proteins. Vaccination increased E6/E7-specific IFNγ^+^ and TNFα^+^ CD8^+^ T-cell response in a dose-dependent manner (Figure 5b–d). Notably, ID immunization produces larger T-cell immune response than IM at the same LNP dose (Figure 5b,d). Consistently, ELISPOT showed that mice immunized by ID exhibited a marked increase in IFNγ secretion versus the IM group at medium (0.2 µg) and high (1 µg) doses, but not at the low dose (0.04 µg) (Figure 5e,f).

### 3.7. Dose-Dependent T Cell Response to ID-Administrated ARV-2001

**Figure 6:**
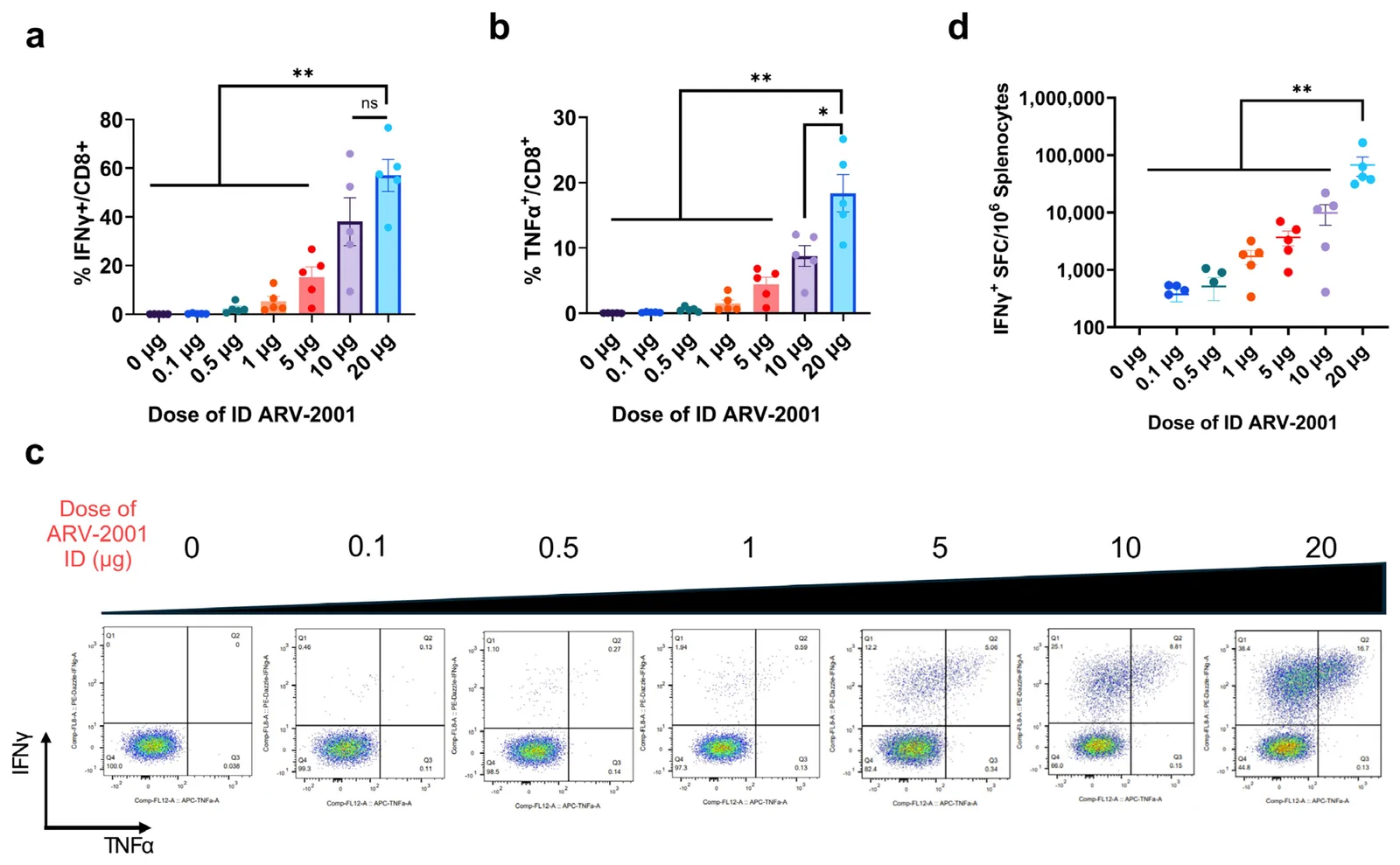
ARV-2001 induces dose-dependent T-cell responses following ID administration. (**a**,**b**) Percentage of IFNγ^+^ (**a**) and TNFα^+^ (**b**) CD8^+^ T cells in the spleen of mice immunized with escalating doses of ARV-2001 by ID injection. (**c**) Representative FACS plots (gated on live CD45^+^CD3^+^CD8^+^) showing IFNγ and TNFα expression in CD8^+^ T cells from ID immunized mice. (**d**) ELISPOT analysis of IFNγ producing cell responses elicited by escalating doses of ARV-2001 administrated by ID. In (**a**,**b**,**d**), each dot indicates an individual mouse. All doses were compared with the 20-µg dose by two-tailed unpaired Student’s *t*-test. ns, not significant; * *p* < 0.05; ** *p* < 0.01.

The superior anti-tumor efficacy was seen with ID administration even at a suboptimal dose (0.5 µg) (Figure 4). However, no plateau in the T-cell response was reached after ID immunization with up to 1 µg ARV-2001 (Figure 5). To further gauge immunogenicity across a wide dose range, we evaluated the T-cell response in naive C57BL/6 mice immunized with 0–20 μg ARV-2001 by ID administration (Figure 6) with a two-dose regimen given 2 weeks apart, as shown in Figure 5a. ID injection induced a dose-dependent T-cell response (IFNγ) measured by both ICS (Figure 6a,c) and ELISPOT (Figure 6d and Figure S10). The TNFα^+^ CD8^+^ T-cell response was likewise dose-dependent (Figure 6b,c). Taken together, ID vaccination elicited a dose-dependent T-cell response to ARV-2001 in C57BL/6 mice.

## 4. Discussion

These studies describe an integrated preclinical rationale for ARV-2001 as a non-surgical immunotherapeutic for HPV-16+ cHSIL. HPV-16 is a uniquely persistent infection amongst the high-risk HPV types, with very low rates of regression [36]. Cervical cell transformation to dysplasia is driven by E6/E7 oncogenes and the persistent expression of these proteins in cervical cells mark the infected and dysplastic/malignant cells make E6/E7 ideal targets for HPV-precancer immunotherapy. Although introduction of E6 and E7 antigens via mRNA containing LNPs into tissues has no chance of transforming cells via persistent, intranuclear expression, other technologies such as vector-delivered immunogens do have this theoretical risk. Thus, we have followed the precedent of several other vaccine platforms that have been tested in past using E6 and E7 with genetically inactivated constructs, abrogating even the theoretical risk that transformation events may occur. The ideal nature of E6/E7 as targets and the wisdom of addressing potential neoplastic cervical disease in the precancer stage has been the basis for multiple attempts to establish immunotherapy [15]. However, the observed regression rates or the quality/powering/controls in the trials have been insufficient to generate compelling data that would drive implementation of this immunotherapy. The proposed use of mRNA/LNPs will, in our view, provide an ideal mechanism for induction of efficacious immunity that should exceed the efficacies previously observed [15]. In addition, we have biased this intervention for further success via innovations that should drive better T-cell killing; the S2 T-helper fusion protein, intradermal delivery, and imiquimod as prime-pull approach each independently demonstrated increased antitumor activity and, importantly, increased effector T-cell infiltration into the tumor. The latter, use of supplemental imiquimod administration may overcome a key limitation of prior therapeutic HPV vaccines, generating circulating E6/E7-specific T cells and augmenting their ability to reach and act within the lesion [12,13].

The mechanistic case for improving historical efficacy rests on platform and delivery biology rather than on target novelty. The dominant prior clinical candidate, the DNA vaccine VGX-3100, required electroporation and relied on cross-presentation for CD8 priming; in its phase 2b study, it produced histologic regression of 49.5% versus 30.6% with placebo, and its subsequent phase 3 program, which adopted a more stringent composite histologic-plus-virologic endpoint, did not lead to approval [14]. By directly loading MHC class I through endogenous translation in APCs and supplying intrinsic LNP adjuvanticity, the mRNA platform generated potent CD8^+^ responses, and ID delivery further increased the CD8 response approximately five-fold over IM while restricting hepatic and systemic exposure [16,20,21]. These properties, combined with helper augmentation and lesional prime-pull, provide a reasonable expectation of improved lesion clearance relative to prior candidates, are to be tested directly in PIN-001.

The biodistribution data are particularly relevant to the target population of reproductive-age women. ID administration localized antigen expression to the skin and draining lymph nodes, minimized hepatic exposure, and yielded only trace, transient ovarian mRNA without detectable systemic E6/E7 protein, a profile consistent with approved mRNA–LNP vaccines and supportive of the reproductive-safety rationale for a cervix-sparing therapy intended to replace LEEP [6,37].

HPV-16 is strictly species-specific and cannot establish productive infection in animals; so, therapeutic HPV vaccine efficacy cannot be evaluated in a natural-infection model. The syngeneic TC-1 model, C57BL/6 cells transformed with HPV-16 E6/E7 and activated H-Ras is the long-standing, regulatory-accepted standard for HPV therapeutic vaccine development because it provides authentic E6/E7-expressing target cells and reads out the same effector mechanism, MHC class I-restricted CD8 killing of E6/E7+ cells, that drives clinical lesion regression [30]. The translational relevance of this readout is supported by the clinical record: multiple programs advanced to the clinic on TC-1-based packages and achieved measurable, T-cell-associated regression, with pooled vaccinated regression of 54% against a 27% placebo background in meta-analysis [15]. We further found that TC-1 reproduces the suppressive immune microenvironment characteristic of human high-grade lesions, the presence of Tregs and MDSCs, mirroring observations in human cHSIL, where regressing lesions are distinguished by low Treg and high intraepithelial CD8 content and where HPV-16-containing lesions in particular show low CD8 and high CD25+ infiltrates and regress least often [38]. The interventions evaluated here (S2 help, imiquimod, ID delivery) act on precisely these axes.

Several limitations are explicitly acknowledged. First, the subcutaneous flank location of TC-1 does not precisely recapitulate the cervicovaginal mucosal microenvironment of human disease. Second, as an aggressive solid tumor, TC-1 models invasive cancer rather than slowly progressing precancer. Although K14-E6/E7 transgenic mice develop HPV-16-driven dysplasia [39], constitutive oncoprotein expression from birth renders E6/E7 self-antigens subject to central tolerance, an immunologically distinct problem from acquired infection in humans; these models are therefore unsuitable for evaluating mRNA immunotherapy directed at foreign E6/E7 antigens. Third, murine MHC restriction means responses are directed against mouse rather than human HLA-restricted epitopes; so, the model cannot validate human epitope coverage. However, several previous clinical trials with other technologies have demonstrated induction of E6/E7 CD8^+^ and T-cells measured against HPV E6 and E7 epitopes, indicating the presence of multiple human-specific HLA-restricted epitopes, suggesting a reasonable expectation of induction of these important effector cells using ARV-2001. Fourth, the model lacks a viral life-cycle component; there is no episomal HPV and therefore no viral-clearance endpoint, although in cHSIL, “clearance” is in practice elimination of E6/E7-expressing cells, the secondary endpoint captured clinically.

The data of TC-1 efficacy modeling were included in an IND submission that was allowed to proceed by the FDA. While acknowledging its limitations, the accumulated clinical experience with this model provides a reasonable expectation that clinical activity will be observed and that the immunological enhancements built into ARV-2001 may improve regression rates relative to prior trials, a hypothesis that PIN-001 is designed to test against an active imiquimod comparator using a pre-specified super-superiority margin.

Dose selection integrated efficacy, immunogenicity, and formulation constraints. We acknowledge that simple body-surface-area or allometric dose scaling is not necessarily appropriate for vaccines or immunotherapies, whose activity depends on innate sensing, antigen expression, injection-site biology, draining lymph-node exposure, species-specific immune responses, and route-specific administration. A 5 µg murine dose, which eradicated established TC-1 tumors, corresponds to approximately 60 µg in humans by allometric scaling [40]; given the absence of an immunogenicity plateau and the formulation ceiling, a 50–75 µg ID dose (100–150 µL) may be evaluated the clinic in future. The first-in-human study PIN-001 will be a randomized, three-arm, open-label phase I/II trial evaluating ARV-2001 with or without topical imiquimod versus imiquimod alone in women aged 25–50 with HPV-16+ cHSIL, with histopathologic clearance at week 28 as the primary efficacy endpoint and HPV-16 clearance and E6/E7-specific T-cell responses (Elispot and flow cytometry) among secondary and immunologic endpoints. The three-arm design directly tests the prime-pull contribution by comparing the combination to ARV-2001 alone, and the embedded immune monitoring is intended to identify a correlate of clearance to support future immunobridging.

## 5. Conclusions

In conclusion, ARV-2001 is a rationally designed mRNA–LNP immunotherapeutic encoding mutated, non-oncogenic HPV-16 E6/E7 fused to a CD4-epitope-rich S2 T-helper domain in a novel ionizable-lipid LNP, delivered intradermally. Preclinically, ARV-2001 is designed to abolish E6/E7 oncogenic activity, while delivering mRNA efficiently, inducing a dose-dependent and route-optimized E6/E7-specific CD8^+^ immunity, and producing robust antitumor efficacy in the TC-1 model. Efficacy is further improved by an imiquimod prime-pull strategy enhancing ingress of T-cells into microenvironment. These data substantiate the mechanism of action proposed for the clinical product and support the future first-in-human evaluation of ARV-2001 as a potential non-surgical, cervix-sparing treatment for HPV-16+ cervical high-grade squamous intraepithelial lesions.

## Data Availability

All datasets generated for this study are included in the article/Supplementary Materials.

## Supplementary Materials

The following supporting information can be downloaded at: https://www.mdpi.com/article/10.3390/vaccines14080714/s1, Figure S1: In vitro expression of the fusion protein from mRNA transfected by Lipofectamine MessengerMAX Transfection Reagent. (a) Graphs showing the relative expression of E6-E7-S2 in each condition normalized with housekeeping protein beta-Actin expression. (b) Representative unprocessed image of target antigen and beta-Actin expressions; Figure S2: Western Blot detection of the E6-E7-S2 fusion protein in ARV-2001 transfected cells. (a) Graphs showing the relative expression of E6-E7-S2 in each condition normalized with housekeeping protein beta-Actin expression. (b) Representative unprocessed image of target antigen and beta-Actin expressions; Figure S3: Western Blot detection of p53 expression. (a) Graphs showing p53 relative expression normalized with non-transfected control. (b) Representative unprocessed image of p53 expressions in different conditions; Figure S4: Gating strategies for immunophenotyping in the tumor microenvironment. Zombie Aqua Fixable viability dye (BioLegend, Cat: 423101) was used to distinguish live cells from dead cells. Antibodies were used as follows: Brilliant Violet™ 421 CD45 (Invitrogen, clone 30-F11, Cat: 404-0451-82), Brilliant Violet 570™ CD11b (BioLegend, clone M1/70, Cat: 101233), Brilliant Violet 605™ CD8 (BioLegend, clone 53-6.7, Cat: 100743), Brilliant Violet 650™ CD25 (BioLegend, clone PC61, Cat: 102038), FITC CD4 (BioLegend, clone RM4-4, Cat: 100406), PE PD-1 (BioLegend, clone 29F.1A12, Cat: 135206), PE/Dazzle™ 594 CD3 (BioLegend, clone 17A2, Cat: 100246), PE/Cyanine7 CD69 (BioLegend, clone H1.2F3, Cat: 104512), Alexa Fluor^®^ 647 FOXP3 (BioLegend, clone MF-14, Cat: 126408), and APC/Cyanine7 Gr-1 (BioLegend, clone RB6-8C5, Cat: 108424); Figure S5: Tumor-infiltrating immune cells induced by ARV-2001 23 days after tumor inoculation in C57BL/6 mice. (a–h) The percentages of CD45^+^, CD8^+^, CD69^+^CD8^+^, PD-1^−^CD8^+^, CD4^+^, CD4^+^CD69^+^, Treg (CD4^+^CD25^+^Foxp3^+^) and CD11b^+^Gr-1^+^ in the tumors from the mice immunized with ARV-2001 and those pre-vaccinated with ARV-1001 followed by ARV-2001 were analyzed by flow cytometry. Mice immunized with ARV-1001 alone and those without immunization were used as controls. *n* = 4–5 mice/group. Statistical analysis using nonparametric Mann–Whitney test, * *p* < 0.05; ns, not significant; Figure S6: TC-1 tumor growth and survival over time after ARV-2001 and IMQ treatment. (a) Mice were immunized 10 days after tumor inoculation, at which point most tumors were palpable. Treatment groups included a suboptimal dose of ARV-2001 alone (IM, 0.5 µg/mouse), IMQ treatment alone (intratumoral injection, 50 µL of 40 µg/ml IMQ in PBS per mouse, corresponding to 100 mg/kg), or the combination of ARV-2001 and IMQ. The combination of ARV-2001 and IMQ significantly inhibited tumor growth (b) and prolonged survival compared to control (c). The combination also improved survival compared to IMQ alone (c). *n* = 5 mice/group. Statistical analysis using ANOVA with Bonferroni post hoc test for (b). Statistical analysis using a log-rank test for (c). * *p* < 0.05; ** *p* < 0.01; *** *p* < 0.001; Figure S7: Tumor-infiltrating immune cells induced by ARV-2001 with or without IMQ. (a–h) The percentages of CD45^+^, CD8^+^, CD69^+^CD8^+^, PD-1^−^CD8^+^, CD4^+^, CD4^+^CD69^+^, Treg (CD4^+^CD25^+^Foxp3^+^) and CD11b^+^Gr-1^+^ in residual tumors from mice immunized with ARV-2001 with or without IMQ were analyzed by flow cytometry. *n* = 5 mice/group of control, IMQ and IMQ+ARV-2001 and *n* = 4 mice/group of ARV-2001. Statistical analysis using nonparametric Mann–Whitney test, * *p* < 0.05; ** *p* <0.01; ns, no significant. Note that one mouse in the ARV-2001 alone group was found dead prior to tumor challenge without having shown any clinical illness; Figure S8: EGFP-Luc mRNA-LNP characterization and in vitro potency. (a) Schematic representation of mRNA encoding EGFP-Luciferase (EGFP-Luc) fusion protein. (b) Physicochemical properties and encapsulation efficiency (EE) of formulated LNP-mRNA. The size and PDI were characterized by dynamic light scattering and EE was measured by a RiboGreen method. (c) The representative overlay images of bright field and GFP fluorescence field of un-transfected control and LNP-mRNA transfected HEK293T cells 24 h post-transfection; Figure S9: The kinetic luminescence at injection sites and livers in mice post-injection of EGFP-Luc mRNA-LNP. BALB/c mice were administered LNP-encapsulated mRNA encoding luciferase at doses of 0.1-(a & d), 1-(b & e), or 5-µg (c & f) via intramuscular (IM, blue) or intradermal (ID, red) injection. Luminescence intensity reflects the efficiency of mRNA delivery and expression in vivo. The values in the region of interest (ROI) each image represent the luminescence intensity. Luciferase expression was monitored at 6-, 24- and 120-h post-injection. The upper panels (a–c) show the expression measured at injection site, and lower panels (d–f) show the signal detected from livers. *n* = 3 mice/group. Statistical analysis using two-tailed unpaired Student’s *t*-test, * *p* < 0.05; *** *p* < 0.001; ns, not significant; Figure S10: Dose-response regression analysis. Dose-response regression analysis was used to evaluate the relationship between doses of ARV-2001 and the numbers of spot-forming cells in Elispot assay.
