# Supplementary material for: Preclinical Development of ARV-2001, an Intradermally Administered mRNA–Lipid Nanoparticle Immunotherapeutic for the Treatment of HPV-16-Positive Cervical High-Grade Squamous Intraepithelial Lesions
Source: Vaccines (Basel). 2026 Aug 19;14(8):714. doi: 10.3390/vaccines14080714 (PMC13517577; doi:10.3390/vaccines14080714)
Supplement: Supplementary file 1 [file vaccines-14-00714-s001.zip › vaccines-4445497-supplementary.pdf]

Supplementary Figures

a

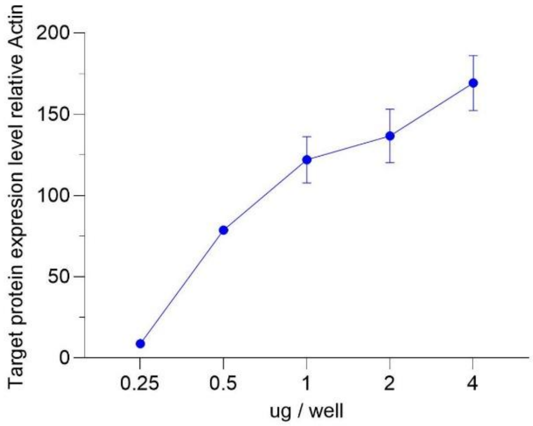

b

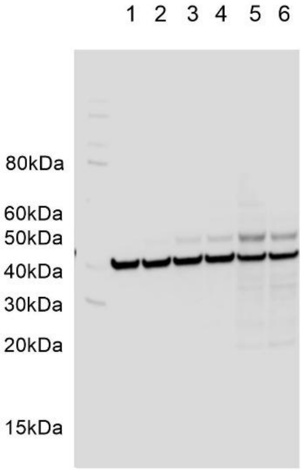

Figure S1. In vitro expression of the fusion protein from mRNA transfected by Lipofectamine MessengerMAX Transfection Reagent. (**a**) Graphs showing the relative expression of E6-E7-S2 in each condition normalized with housekeeping protein beta-Actin expression. (**b**) Representative unprocessed image of target antigen and beta-Actin expressions.

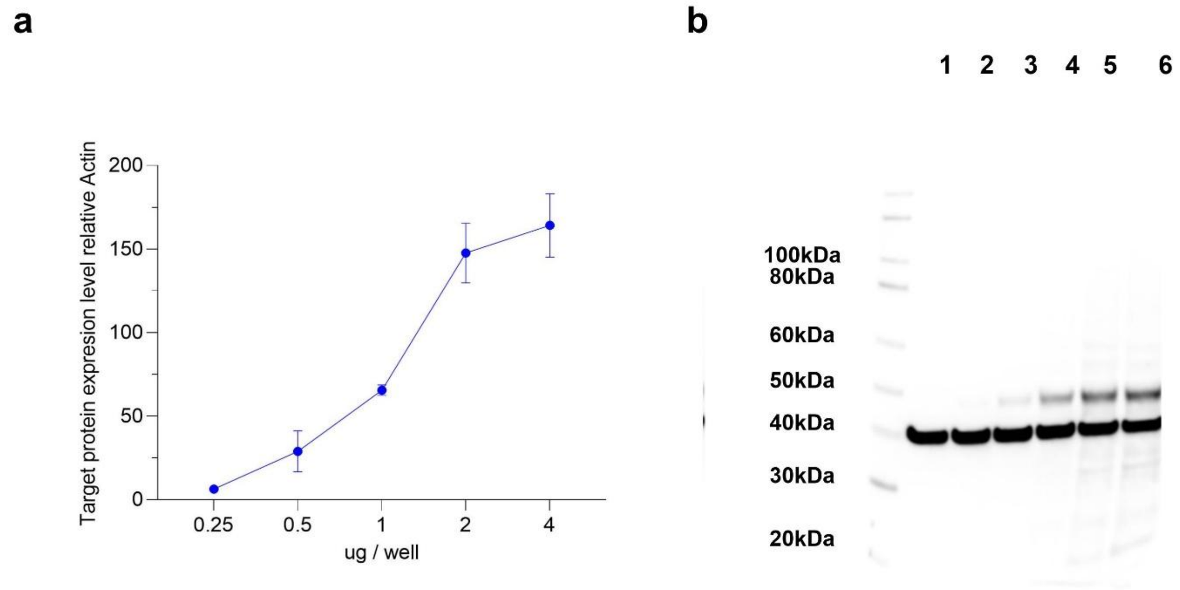

Figure S2. Western Blot detection of the E6-E7-S2 fusion protein in ARV-2001 transfected cells. (**a**) Graphs showing the relative expression of E6-E7-S2 in each condition normalized with housekeeping protein beta-Actin expression. (**b**) Representative unprocessed image of target antigen and beta-Actin expressions.

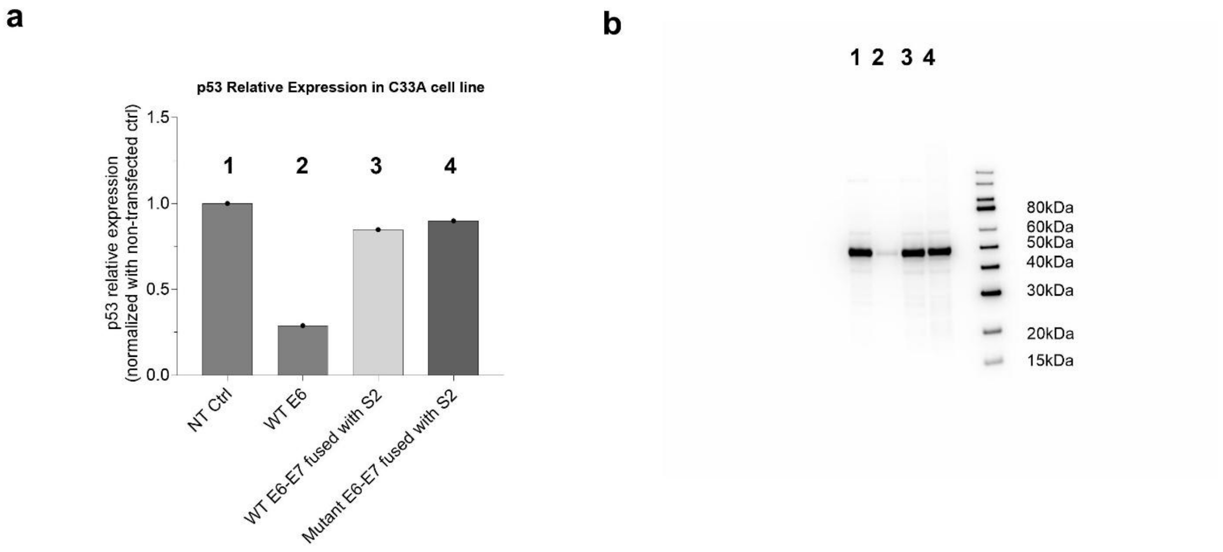

Figure S3. Western Blot detection of p53 expression. (**a**) Graphs showing p53 relative expression normalized with non-transfected control. (**b**) Representative unprocessed image of p53 expressions in different conditions.

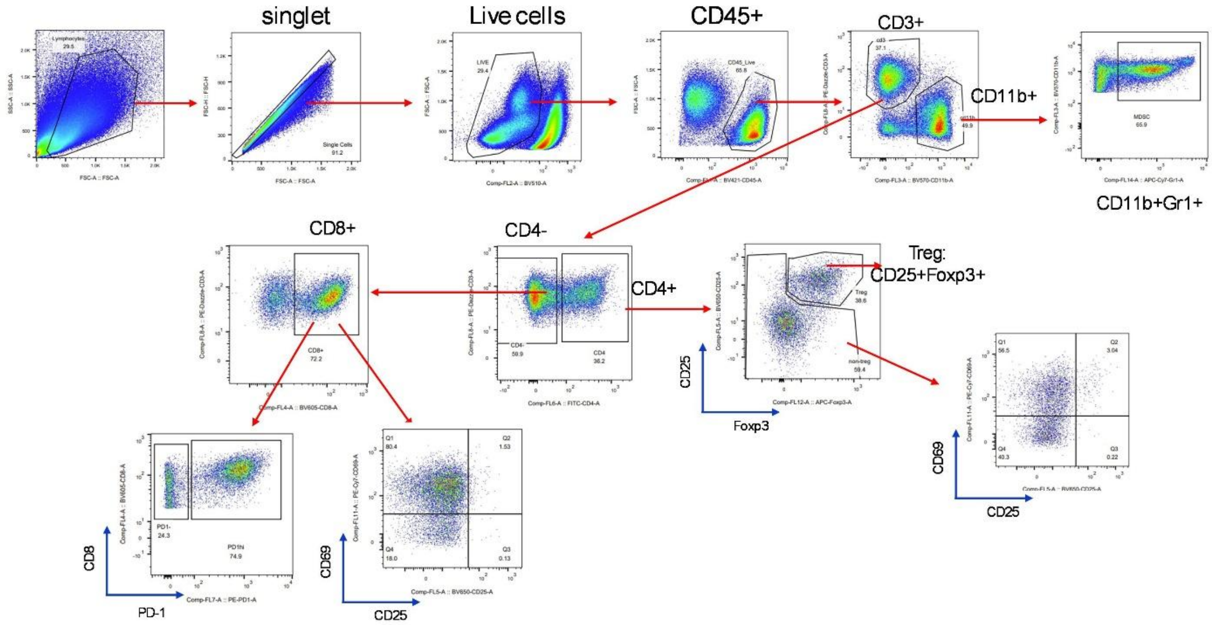

Figure S4. Gating strategies for immunophenotyping in the tumor microenvironment. Zombie Aqua Fixable viability dye (BioLegend, Cat: 423101) was used to distinguish live cells from dead cells. Antibodies were used as follows: Brilliant Violet™ 421 CD45 (Invitrogen, clone 30-F11, Cat: 404-0451-82), Brilliant Violet 570™ CD11b (BioLegend, clone M1/70, Cat: 101233), Brilliant Violet 605™ CD8 (BioLegend, clone 53-6.7, Cat: 100743), Brilliant Violet 650™ CD25 (BioLegend, clone PC61, Cat: 102038), FITC CD4 (BioLegend, clone RM4-4, Cat: 100406), PE PD-1 (BioLegend, clone 29F.1A12, Cat: 135206), PE/Dazzle™ 594 CD3 (BioLegend, clone 17A2, Cat: 100246), PE/Cyanine7 CD69 (BioLegend, clone H1.2F3, Cat: 104512), Alexa Fluor® 647 FOXP3 (BioLegend, clone MF-14, Cat: 126408), and APC/Cyanine7 Gr-1 (BioLegend, clone RB6-8C5, Cat: 108424).

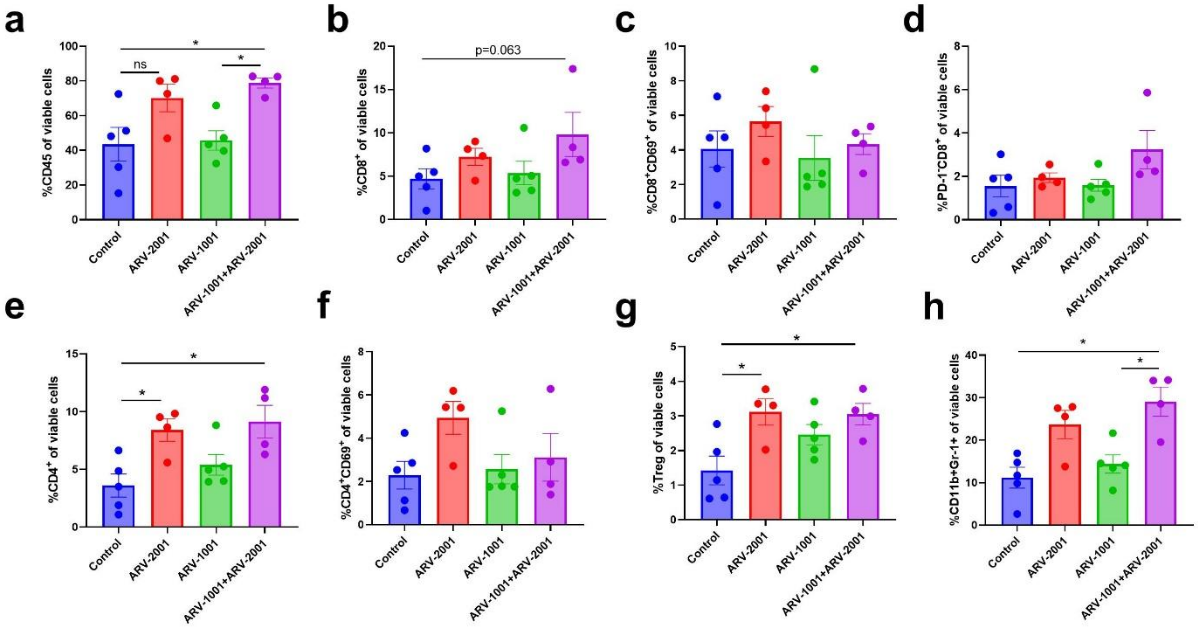

Figure S5. Tumor-infiltrating immune cells induced by ARV-2001 23 days after tumor inoculation in C57BL/6 mice. (**a-h**) The percentages of CD45$^+$, CD8$^+$, CD69$^+$CD8$^+$, PD-1$^-$CD8$^+$, CD4$^+$, CD4$^+$CD69$^+$, Treg (CD4$^+$CD25$^+$Foxp3$^+$) and CD11b$^+$Gr-1$^+$ in the tumors from the mice immunized with ARV-2001 and those pre-vaccinated with ARV-1001 followed by ARV-2001 were analyzed by flow cytometry. Mice immunized with ARV-1001 alone and those without immunization were used as controls. n = 4-5 mice/group. Statistical analysis using nonparametric Mann–Whitney test, * $p < 0.05$; ns, not significant.

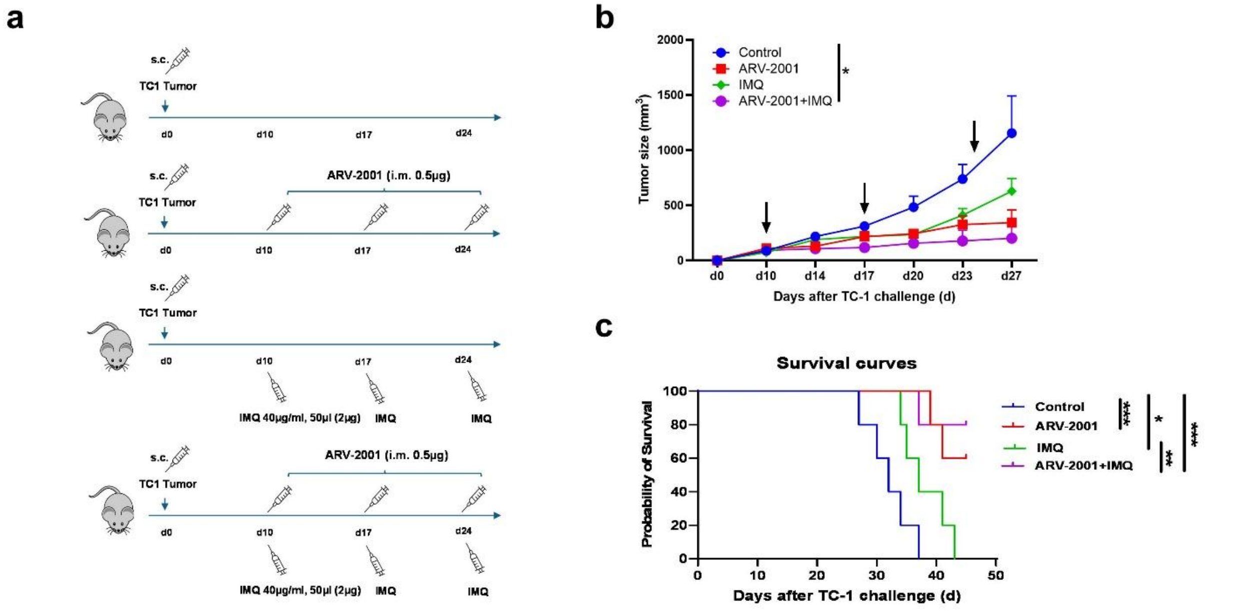

Figure S6. TC-1 tumor growth and survival over time after ARV-2001 and IMQ treatment. (**a**) Mice were immunized 10 days after tumor inoculation, at which point most tumors were palpable. Treatment groups included a suboptimal dose of ARV-2001 alone (IM, 0.5μg/mouse), IMQ treatment alone (intratumoral injection, 50 μl of 40 μg/ml IMQ in PBS per mouse, corresponding to 100mg/kg), or the combination of ARV-2001 and IMQ. The combination of ARV-2001 and IMQ significantly inhibited tumor growth (**b**) and prolonged survival compared to control (**c**). The combination also improved survival compared to IMQ alone (**c**). *n* = 5 mice/group. Statistical analysis using ANOVA with Bonferroni post hoc test for (**b**). Statistical analysis using a log-rank test for (**c**). * $p < 0.05$; ** $p < 0.01$; *** $p < 0.001$.

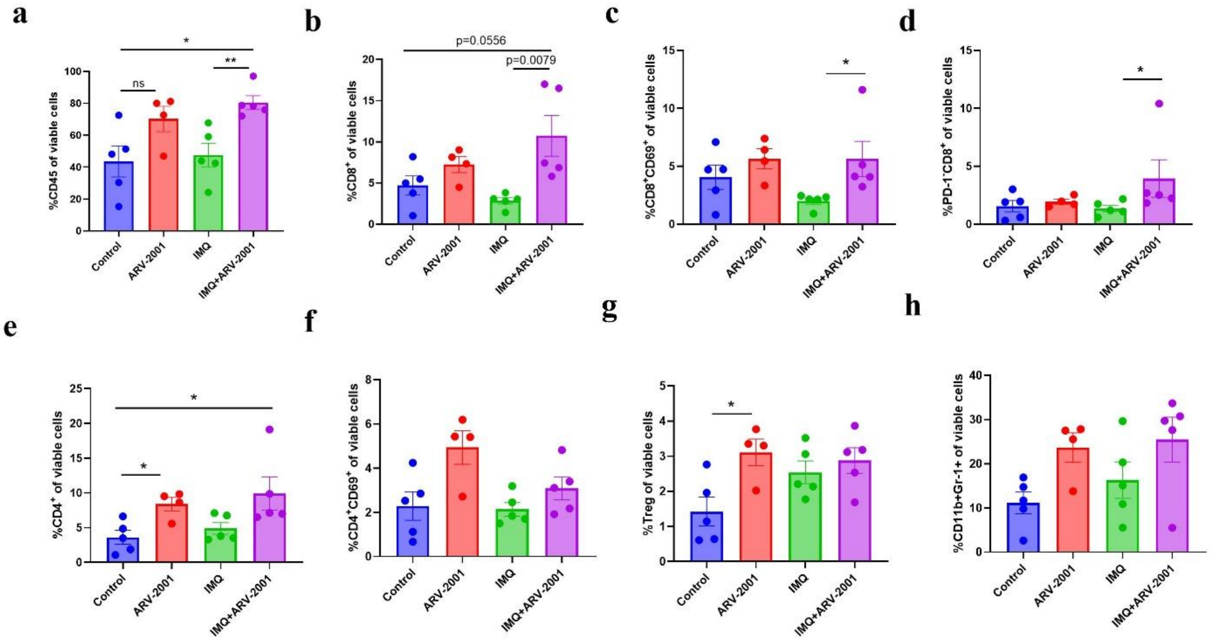

Figure S7. Tumor-infiltrating immune cells induced by ARV-2001 with or without IMQ. (**a-h**) The percentages of CD45$^+$, CD8$^+$, CD69$^+$CD8$^+$, PD-1$^-$CD8$^+$, CD4$^+$, CD4$^+$CD69$^+$, Treg (CD4$^+$CD25$^+$Foxp3$^+$) and CD11b$^+$Gr-1$^+$ in residual tumors from mice immunized with ARV-2001 with or without IMQ were analyzed by flow cytometry. n = 5 mice / group of control, IMQ and IMQ+ARV-2001 and n = 4 mice / group of ARV-2001. Statistical analysis using nonparametric Mann–Whitney test, * p < 0.05; ** p <0 .01; ns, no significant. Note that one mouse in the ARV-2001 alone group was found dead prior to tumor challenge without having shown any clinical illness.

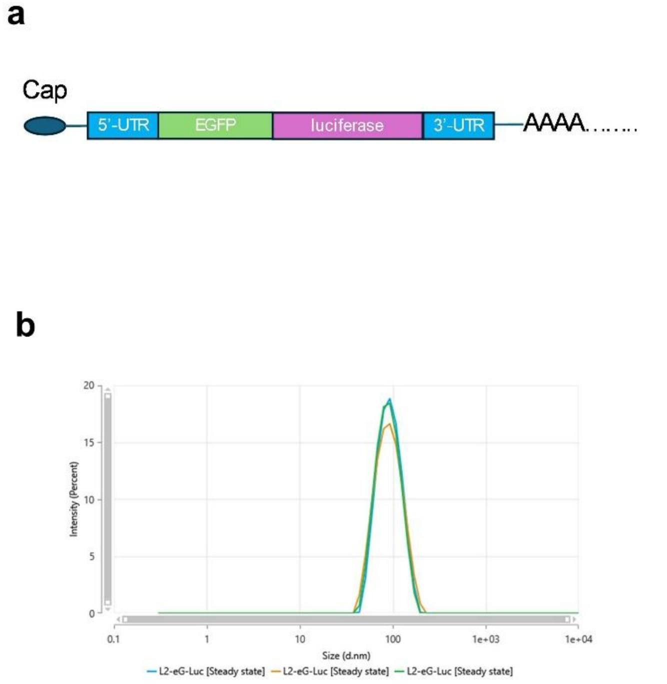

Sample	Size(nm)	PDI	EE(%)
EGFP-luciferase	85.96	0.07552	91.9

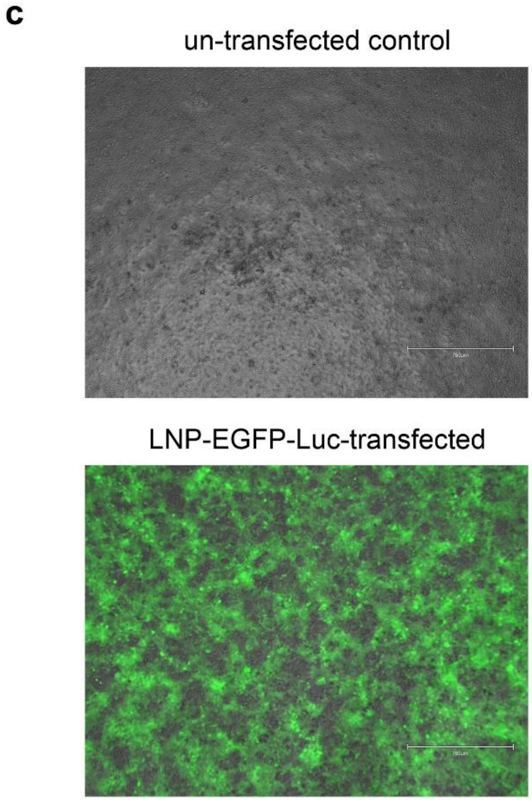

Figure S8. EGFP-Luc mRNA-LNP characterization and in vitro potency. (**a**) Schematic representation of mRNA encoding EGFP-Luciferase (EGFP-Luc) fusion protein. (**b**) Physicochemical properties and encapsulation efficiency (EE) of formulated LNP-mRNA. The size and PDI were characterized by dynamic light scattering and EE was measured by a RiboGreen method. (**c**) The representative overlay images of bright field and GFP fluorescence field of un-transfected control and LNP-mRNA transfected HEK293T cells 24 hr post-transfection.

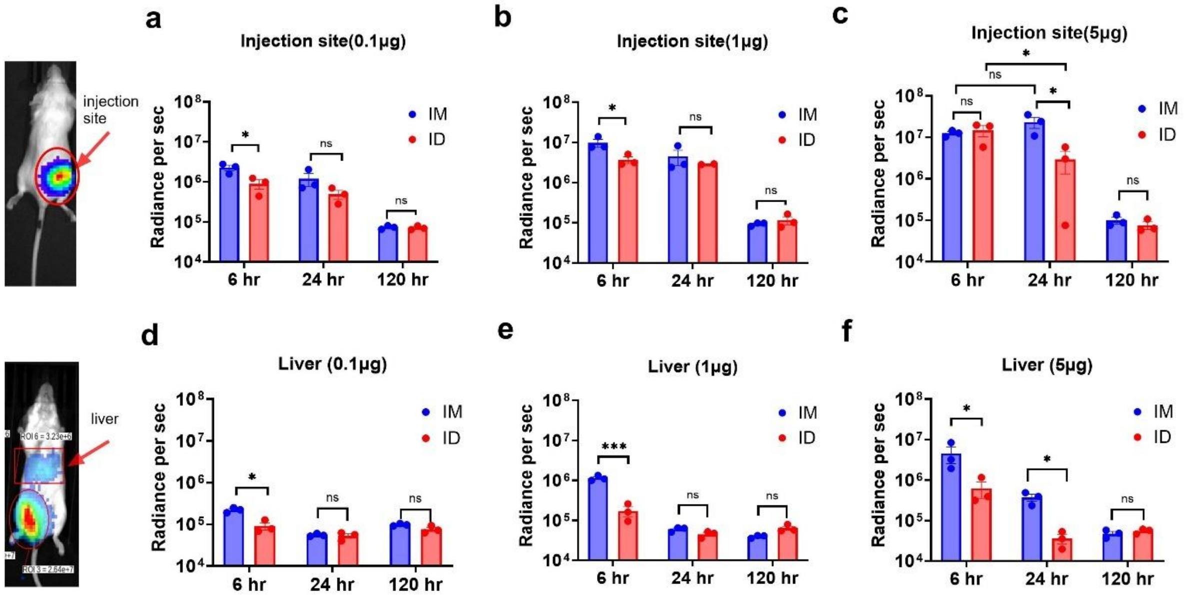

Figure S9. The kinetic luminescence at injection sites and livers in mice post-injection of EGFP-Luc mRNA-LNP. BALB/c mice were administered LNP-encapsulated mRNA encoding luciferase at doses of 0.1-(**a** & **d**), 1- (**b** & **e**), or 5- µg (**c** & **f**) via intramuscular (IM, blue) or intradermal (ID, red) injection. Luminescence intensity reflects the efficiency of mRNA delivery and expression in vivo. The values in the region of interest (ROI) each image represent the luminescence intensity. Luciferase expression was monitored at 6-, 24- and 120-hrs post-injection. The upper panels (**a-c**) show the expression measured at injection site, and lower panels (**d-f**) show the signal detected from livers. *n* = 3 mice/group. Statistical analysis using two-tailed unpaired Student's t-test, * $p < 0.05$; *** $p < 0.001$; ns, not significant.

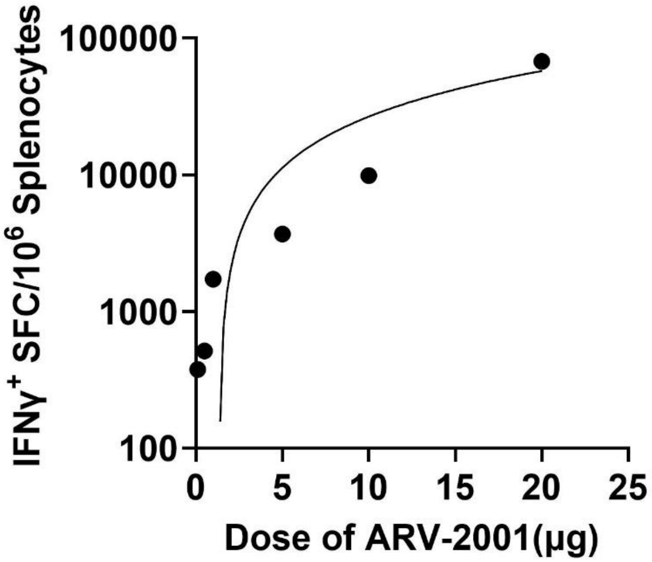

Figure S10. Dose-response regression analysis. Dose-response regression analysis was used to evaluate the relationship between doses of ARV-2001 and the numbers of spot-forming cells in Elispot assay.
